# Mesenchymal Stem Cells for Cardiac Regeneration: Translation to Bedside Reality

**DOI:** 10.1155/2012/646038

**Published:** 2012-06-07

**Authors:** Mohammad T. Elnakish, Fatemat Hassan, Duaa Dakhlallah, Clay B. Marsh, Ibrahim A. Alhaider, Mahmood Khan

**Affiliations:** ^1^Department of Physiology and Cell Biology, The Dorothy M. Davis Heart & Lung Research Institute, The Ohio State University, Columbus, OH 43210, USA; ^2^Division of Cardiovascular Medicine, Department of Internal Medicine, The Dorothy M. Davis Heart & Lung Research Institute, The Ohio State University, Columbus, OH 43210, USA; ^3^Division of Pulmonary and Critical Care, Department of Internal Medicine, The Dorothy M. Davis Heart & Lung Research Institute, The Ohio State University, Columbus, OH 43210, USA; ^4^Department of Pharmaceutical Sciences, College of Clinical Pharmacy, King Faisal University, Al-Ahsa 31982, Saudi Arabia

## Abstract

Cardiovascular disease (CVD) is the leading cause of death worldwide. According to the World Health Organization (WHO), an estimate of 17.3 million people died from CVDs in 2008 and by 2030, the number of deaths is estimated to reach almost 23.6 million. Despite the development of a variety of treatment options, heart failure management has failed to inhibit myocardial scar formation and replace the lost cardiomyocyte mass with new functional contractile cells. This shortage is complicated by the limited ability of the heart for self-regeneration. Accordingly, novel management approaches have been introduced into the field of cardiovascular research, leading to the evolution of gene- and cell-based therapies. Stem cell-based therapy (aka, cardiomyoplasty) is a rapidly growing alternative for regenerating the damaged myocardium and attenuating ischemic heart disease. However, the optimal cell type to achieve this goal has not been established yet, even after a decade of cardiovascular stem cell research. Mesenchymal stem cells (MSCs) in particular have been extensively investigated as a potential therapeutic approach for cardiac regeneration, due to their distinctive characteristics. In this paper, we focus on the therapeutic applications of MSCs and their transition from the experimental benchside to the clinical bedside.

## 1. Introduction

Ischemic heart disease and congestive heart failure together are identified as the leading cause of death worldwide [1]. Myocardial infarction (MI, aka heart attack) occurs as a result of cardiomyocytes death leading to loss of viable myocytes, which lack endogenous repair mechanisms. If left untreated, it will lead to fibrous scar formation replacing the damaged myocardium with subsequent congestive heart failure (CHF) [2]. Despite, the development of a wide array of treatment options, heart failure management has failed to replace the lost cardiomyocyte mass with new contractile cells. The main challenge facing treatment options is the limited ability of the heart for self-regeneration [3]. This led to the introduction of gene- and cell-based therapeutic approaches to treat the damaged heart [4].

In an attempt to replace cardiomyocytes lost after ischemia, cellular therapy/cardiomyoplasty has been rigorously investigated in the last few years due to the potential benefits in patients with a variety of cardiac diseases such as acute MI, stable coronary artery disease, and heart failure [5]. The goals of cell-based therapies for cardiac diseases are reliant on the primary pathology, whether it is myocardial ischemia, cardiac dysfunction, or both. In myocardial ischemia, cellular transplantation is expected to provide a renewable source of proliferating, functional cardiomyocytes and simultaneously trigger neovascularization in order to provide a novel network of blood vessels to support and nourish the newly forming cardiomyocytes [4]. Experimental evidence has recognized numerous stem, progenitor, and mature cells that can induce these mechanisms *in vivo*, including embryonic stem cells (ESCs), unfractionated bone marrow cells (BMCs) and mononuclear cells (BMMNCs), hematopoietic stem cells (HSCs), mesenchymal stem cells (MSCs), endothelial progenitor cells (EPCs), cardiac progenitor cells, skeletal myoblasts, fetal cardiomyocytes, and induced pluripotent stem cells (IPSCs) [6].

Embryonic stem cells (ESCs) are derived from the inner mass of the developing embryo during the blastocyst stage. Being the prototypical stem cell, these cells have exhibited the highest potential for organ regeneration including the heart [7, 8]. Recently, it has been reported that ESCs can differentiate into cardiac precursor cells and stimulate myocyte development [9]. On the other hand, their native propensity for pluripotent proliferation increased the risk of teratoma formation [10]. Another potential challenge for their clinical use is immunological incompatibility as a result of their allogenic origin [11]. In addition, some social and ethical concerns have been raised due to the methods by which they are obtained [8].

Unlike pluripotent embryonic stem cells, adult stem cells exhibit a limited capability of differentiation. The bone marrow represents a classic adult stem cell source, containing diverse cell populations (e.g., HSCs, EPCs and MSC) that are able to migrate and transdifferentiate into distinct phenotypes. However, the ability of these cells to differentiate into cardiac myocytes is indecisive [12–15]. Additionally, hematopoietic stem cells (HSCs) are normally recognized by the expression of CD34^+^ and CD133 cell surface markers. HSCs have been broadly investigated and successfully used clinically for bone marrow transplantation in a variety of hematologic disorders [16]. On the other hand, endothelial progenitor cells (EPCs) represent a heterogeneous population of cells that mainly exist in the bone marrow (BM). These cells are thought to induce neovascularization, possibly playing a vital role in vascular homeostasis and even myogenesis [17].

Skeletal myoblasts (SM) were the first cells to evolve into clinical trials and injected into the ischemic myocardium [18]. Despite the great potential that these cells had on MI patients, the clinical trial was shut down due to the development of serious ventricular arrhythmias in the myoblast-injected hearts [19]. Even though enhancements in left ventricular (LV) function and volumes were reported, they were not sustained [20–22]. Cardiac stem cells or progenitor cells are the other cell types that have been identified in human and mammalian hearts; these cells can be obtained from surgical or endomyocardial biopsies and clonally expanded *in vitro.* The exact origin of these cells whether intracardiac or extracardiac is unknown and needs to be precisely determined by lineage tracing experiments [23–27]. These cells exhibit a high proliferative potential, but this does not seem to be sufficient to heal extensive injuries as that of MI [28, 29]. Recently a novel population of stem cells, known as induced pluripotent stem cells (iPSCs), with the characteristic properties of embryonic stem cells (ESCs) but derived from regular somatic cells such as adult fibroblasts were discovered. These human-stimulated pluripotent stem cells are developed through nuclear reprogramming, transduction of stemness factors, and the ectopic expression of pluripotency genes into fibroblasts [30–35]. This innovative approach offers an alternative source of stem cell lines with cardiogenic potential without the conflicts of using eggs or embryos [16]; however the clinical applications need to be further established [36, 37].

As described above stem cell-based therapy displays exciting promises for regenerating the damaged myocardium and treating heart failure. However, the optimal cell type to achieve this goal needs to be further investigated. MSCs, due to their distinctive characteristics properties, have been extensively investigated as an appealing therapeutic approach for cardiac regeneration. In this paper we will focus on the therapeutic applications of MSCs and their transition from the experimental benchside to the clinical bedside.

## 2. Mesenchymal Stem Cells

In the 1970s, Friedenstein et al. showed that the bone marrow contains a population of HSCs and an infrequent population of stromal cells, which are now known as mesenchymal stem cells (MSCs) [38]. They were the earliest researchers to display the capability of MSCs to differentiate into mesoderm-derived tissue and to recognize their significance in regulating hematopoiesis [39]. In the 1980s, different research groups further established that MSCs can differentiate into osteoblasts, chondrocytes, and adipocytes [40, 41]. Later in the 1990s, Wakitani et al. demonstrated that MSCs can differentiate into a myogenic phenotype [42]. In 1999, Kopen et al. revealed that MSCs are even able to transdifferentiate into ectoderm-derived tissue [43]. In the same year, Makino et al. reported for the first time the ability of mouse BM-derived MSCs to specifically form cardiomyocytes *in vitro* [44], and later on Toma et al. showed the same findings *in vivo* [45].

Furthermore, MSCs also exert immunomodulatory effects, and they do not elicit an immune response on allogenic transplantation due to the inhibition of T-cell proliferation [46]. MSCs are shown to express HLA (human leukocyte antigen) class I, but not HLA class II on their cell surface membrane [47]. Undifferentiated as well as differentiated MSCs do not show proliferative lymphocytic immune responses upon allogenic transplantation [47]. MSCs have also been recognized for their possible role in prophylaxis and treatment of graft versus host disease [48, 49]. The immunomodulatory properties of MSCs further permit them for their clinical large-scale production and allogenic transplantation [50].

### 2.1. Sources, Definitions, and Types of Mesenchymal Stem Cells

MSCs have been identified in almost every tissue type such as brain, spleen, liver, kidney, lung, BM, muscle, skin, adipose tissue, thymus, aorta, vena cava, and pancreas of adult mice. MSCs might be located in all postnatal organs [51, 52]; yet the most abundant source is the BM [53]. So far, there is no exact definition for MSCs; consequently, MSCs are generally defined functionally, rather than by the existence of specific surface markers [53]. MSCs adhere to cell culture dishes without expressing the surface markers that distinguish the HSCs [54]. Despite the variation in characterizing a particular phenotype among different studies, it is normally accepted that MSCs are negative for CD11b, CD14, CD31, CD34, and CD45. However, they are positive for CD29, CD44, CD73, CD105, CD106, and CD166 [2, 55–58]. MSCs are an infrequent population in the BM, representing about 0.001–0.01% of total nucleated cells [59]. In culture they possess a spindle-shaped fibroblast-like appearance and the capability of expanding noticeably in culture, sustaining their multilineage potential [2]. These adherence criteria in culture and potential of multilineage are the most frequently established definitions of MSCs [53].

MSCs comprise several subpopulations, including, recycling stem (RS) cells, multipotent adult progenitor cells (MAPCs), human BM-derived multipotent stem cells (hBMSCs), and cardiac stem cells known as cardiac stromal cells (CStCs). Recycling stem (RS) cells represent the smallest, highly dividing group of MSCs and are thought to be the more primitive form [53]. Unlike MSCs, RS cells do not express hematopoietic stem cell surface markers, but they are unique compared to other MSCs in expressing the stem cell factor receptor (c-kit) [60]. The multipotent adult progenitor cells (MAPCs) are distinct from other MSCs in being immortal in culture. MAPCs share with human BM-derived multipotent stem cells (hBMSCs) their ability to produce cell types from all three germ layers [61, 62]. hBMSCs were shown to engraft and differentiate to multiple lineages in a rodent model of postinfarcted heart failure [63]. Recently, a trend towards using tissue specific stem cells has led to the identification of a novel type of cardiac stem cells known as cardiac stromal cells (CStCs) [64]. Rossini et al. were able to exhibit the differentiation abilities of these CStCs and the conventionally used bone-marrow-derived MSCs [64]. In this study, they showed that despite the fact that CStCs were less able to acquire the osteogenic and adipogenic phenotypes, they were able to express cardiovascular markers more efficiently. Moreover, CStC showed longer survival of transplanted cells into the infarcted heart and better ability to differentiate into cardiomyocytes than bone-marrow-derived MSCs [64].

### 2.2. Therapeutic Applications of Mesenchymal Stem Cells in Cardiac Regeneration

During the last decade, there has been growing interest in MSCs as a therapeutic approach for treating MI, in comparison with the other cell types considered for cardiomyoplasty. MSCs have exclusive properties that may translate into convenient and extremely effective cell therapy [2]. MSCs can be easily isolated with a high expansion potential in culture providing the large numbers of cells required for transplantation within a short period of time. Their characteristic properties include the following: (1) genetic stability, (2) compatibility with tissue engineering principles, (3) reproducibility of features between different bone marrow isolates, (4) their potential to trigger regeneration in various fundamental tissues including the myocardium and neovascularization, (5) they have the ability to home to the damaged tissue or inflammatory sites, and (6) moreover their immunoregulatory properties could allow their use as an allogenic treatment. MSCs can be delivered systemically, for example, via IV injection, which simplifies administration without the necessity for cardiac catheterization laboratories. These cells can be readily transduced by a range of vectors and retain transgene expression after *in vivo* differentiation, which might be used eventually to enhance cell engraftment or the degree of differentiation [2, 4, 53].

## 3. Mesenchymal Stem Cell Therapy: Benchside

In the field of MSC transplantation into cardiac tissue, animal models mostly focus on fate, efficacy, regenerative mechanisms, and the safety of transplanted MSCs. In line with the increased incidence of myocardial infarction, both small and large animal models have been used in large numbers, providing the proof of functional effectiveness, pathomechanisms, and safety of MSC transplantation [65]. In this context, it has been reported that BM cells were used for the first time for cardiomyoplasty in 1999 by the laboratories of  Tomita et al. [66]. In this paper, rats received autologous BMCs via direct intramyocardial injection at 3 weeks after cryoinjury. Eight weeks postcryoinjury researchers were able to identify transplanted BMCs in all animals. They found that these cells expressed muscle-specific proteins that were absent prior to implantation. Moreover, they reported improvements in systolic and diastolic functions in animals that received cells pretreated with the DNA-demethylating agent 5-azacytidine (5-Aza), which has been established to augment myogenic differentiation of pluripotent stem cells [44]. Thereafter, numerous preclinical studies reported improvement of left ventricular (LV) function, decreased infarct size, and decreased mortality rate after transplantation of MSCs in mice [65, 67–71], rats [72–79], swine [80–92], canine [93, 94], and sheep [78, 95] after acute or chronic MI. These enhancements were observed even with minimal percentage of cells exhibiting cardiomyocytes differentiation [65, 70, 94] (Table 1).

Due to the anatomical similarity to the human heart, swine heart has been chosen as a model for studies related to MI and general cardiovascular studies [96]. This model has been used to acquire significant information on the tracking of transplanted MSCs in healthy and infracted myocardium and the immediate and long-term effects after engraftment [97]. In the swine model, Shake et al. reported strong engraftment of labeled MSCs along with coexpression of numerous muscle-specific proteins as early as two weeks after intramyocardial implantation. This study proposed that the differentiation of MSCs into cardiomyocyte-like cells occurs two weeks after transplantation, followed by a significant improvement of contractile dysfunction and wall thinning [92]. A similar study by Schuleri et al. showed that intramyocardial transplantation of MSCs resulted in a significant increase of LV function eight weeks after transplantation [90]. These improvements were preceded by an early enhancement of resting myocardial blood flow after one week, which was confirmed by an increase in vessel size in the MSC group versus the control groups. These observations suggest that transplantation of MSCs can ameliorate cardiac function by reducing infarct size, triggering neovascularization and cardiomyogenesis (Table 1). 

The optimization of safety and possible procedures for cell delivery are central issues to be considered in cardiomyoplasty. By using large animal models (i.e., swine, dogs, sheep), the majority of investigators have revealed that the intramyocardial injection of progenitor cells across the infarcted region is safe and possible [97]. For example, experiments on the swine model showed that intramyocardial injection of MSCs (range: 10^4^–10^8^ cells) is safe and does not result in any obvious immune or toxic response [92, 96, 98–100]. On the other hand, studies on dose-dependent effects have displayed no relevant results to date [84]. Also, “off the shelf” application of allogenic MSCs in a swine safety study with repeated intramyocardial injections of high doses of MSCs (up to 800 × 10^6^ cells) was devoid of adverse effects in terms of sustained ventricular arrhythmia, anaphylaxis, or myocardial damage [101]. Additionally, the procedural safety of the intramyocardial injection process was demonstrated in a canine chronic ischemia model. Dogs that received intramyocardial injections of MSCs (1 × 10^8^ total cells) tolerated the procedure without exhibiting any complications such as cardiac arrhythmias or myocardial damage [94].

On the other hand, intravenous infusion of MSCs in swine changed the electrophysiological properties of the myocardium [87]. In this study, there was significant increase in cardiac function and decrease in eccentric hypertrophy; however, there was also a shortening in epicardial effective refractory periods in MSC-treated animals in comparison with placebo. Shortened effective refractory periods might trigger ventricular tachycardia [102] and increase the possibility of MSCs to trigger proarrhythmic remodeling. In contrast to these observations in swine, intravenous infusion of allogenic MSCs in humans with acute MI revealed fewer ventricular arrhythmias than in those with placebo infusion [103]. These studies revealed that intravenous allogenic MSCs are safe in patients with acute MI. Likewise, MSC therapy in other clinical trials was not associated with any adverse effects [104, 105].

In addition to cardiac arrhythmia and myocardial damage, a number of reports have raised concerns about tumor formation as a result of using BM-cultured MSCs. In these reports murine-derived BM-MSCs exhibited chromosomal abnormalities that led to tumor formation in many organs [106, 107]. In addition, a recent report revealed that both MSCs and BM-derived stem cells have been associated with calcification and probably ossification of the heart in a murine model of MI [108]. In contrast to these observations, numerous large-animal preclinical studies displayed the safety of MSCs therapy and are devoid of tumor formation or ectopic tissue growth [80, 81, 83–87, 90, 91, 94]. Moreover, data from early-phase human studies using MSCs showed no evidence of ectopic tissue growth [103–105]. Even so, the data of tumorigenesis in murine models emphasizes the necessity of persistent long-term monitoring of patients treated with MSCs. Furthermore, other reports have shown that intracoronary injection of MSCs in canine and swine models of MI resulted in microinfarctions and slow coronary arterial flow, respectively [109, 110]. Microvascular obstruction with intracoronary MSCs injection may be explained by the fact that the size of MSCs is larger than other stem cell types and their characteristic adherence to plastic *in vitro* [53]. Nonetheless, this does not appear to be a problem in the limited clinical experience, so far [53].

In summary, MSC therapy has been shown to be safe and effective in improving LV function, decreasing scar size, and increasing myocardial tissue perfusion and angiogenesis in post-MI small and large animal models. Yet, it is hard to evaluate the impact of these preclinical studies on MI patients. In regard to effectiveness of MSCs, data displaying a time-dependent retention, engraftment, migration, and differentiation support the notion that MSC implantation is an alternative therapeutic approach for ischemic heart failure [97]. Considering procedural safety, it could be presumed that the reliable security findings displayed by the swine studies may be applicable to humans [111]. Nevertheless, it is clear that further studies are needed.

### 3.1. Modification of Mesenchymal Stem Cells for Cardiac Therapy

Regardless of the benefits of MSCs, clinical application of MSC-based therapy is restricted. This restriction is attributed to the poor viability of the transplanted cells in the myocardium [29]. Recent reports on a swine model of MI displayed that only 5% of implanted MSCs can survive for 14 days in the infracted myocardium [109]. In addition, Toma et al. showed that the survival rate of the implanted hMSCs in an intact mouse heart is less than 0.5% at 4 days after transplantation [45]. Analogous outcomes were also obtained from studies using diverse cell types. Accordingly, cell viability posses a major obstacle for any cell-based therapeutic strategy in the infarct heart [29]. Secondly, reactive oxygen species (ROS) is known to be a key mediator in cardiac dysfunction. ROS is known to hinder cell adhesion and stimulate cell detachment and death [112–115]. Third, the grafted cell may encounter ischemic conditions lacking nutrients and oxygen and consequently affecting cell viability [116, 117]. On the other hand, myocardial injury has been shown to generate a strong inflammatory response followed by production of oxygen-derived free radicals and inflammatory cytokines that trigger cell death and initiate apoptosis [118]. Despite all these, MSCs may react differently in the allogenic settings due to their previously described immunomodulatory effects on inflammatory cells [29].

**Figure 1:**
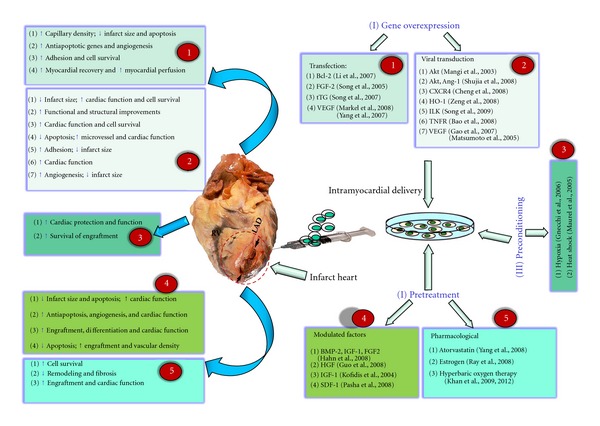
Illustration of MSC modifications and its effect after transplantation on engraftment, cell survival, apoptosis, cardiac function, fibrosis, and angiogenesis in animal models of MI.

To overcome the low cellular survival and transdifferentiation strength of MSCs after transplantation, several strategies have been proposed for MSCs manipulation (Figure 1). Pretreatment with growth and differentiation factors to expand the stem cells and facilitate their engraftment into cardiac tissues has been attempted [25, 119–121]. Also pretreatment with pharmacological agents such as estrogen, which influences myocardial remodeling through stimulating growth hormone production in BM-MSCs and EPCs [122] or through atorvastatin which enhances cell survival and differentiation into cardiomyocytes [123]. More recently, our group has demonstrated that combined treatment of rats with stem cells and pharmacological hyperbaric oxygen (HBO) treatment led to enhanced cell engraftment and decreased fibrosis at four weeks after transplantation [55, 124]. Furthermore, stem cell preconditioning prior to transplantation, such as hypoxic preconditioning, has been shown to activate the Akt signaling pathway and the heat shock protein (Hsp-70), therefore, maintaining cell viability and cell cycle rates [125, 126]. Moreover, overexpression of anti-cell-death signals or signals that improve cell adhesion resulted in better recovery and adhesion after transplantation [76, 77, 127–137]. 

Additionally, recent studies have shown that microRNAs (miRNAs) are one of the key modulators in stem cell differentiation. MiRNAs regulate gene expression in stem cells that control its fate, function, and behavior. The most important change in ESCs by miRNAs is the cell differentiation, it was shown that *miR-21*,* miR-134*, and *miR-470* target certain genes to promote cell differentiation [138–140]. At the same time, stem cell transcription factors and silencing complexes bind to miRNAs promoter region and regulate their expression during early cellular differentiation. In murine ESCs, the most abundant miRNAs was *miR-17-92* cluster and *miR-302* that have been key regulators of cellular proliferation [141–143]. It is worth mentioning that these miRNAs are involved in maintaining DNA methylation and facilitating repression and overexpression of certain genes through differentiation and development [144]. Recent findings have shown that *miR-150* regulates the mobilization and migration of bone marrow mononuclear cells by targeting CXCR4 [145]. 

## 4. Mesenchymal Stem Cell Therapy: Bedside

For any particular type of cell-based therapy to be translated from the preclinical benchside to the clinical bedside, Murry et al. [146] proposed specific criteria; first preclinical revelation of safety and efficacy should be evident reproducibly in manifold laboratories. The inability of professionals in a well-controlled laboratory to reproduce certain finding means that the probability of such treatment to succeed in the more capricious world of human clinical trials is low, understanding the mechanisms of action to a logical extent is also necessary, and especially the mechanisms by which cell therapy causes functional improvements will help in designing rational experimental and/or clinical studies to improve the treatment effectiveness. Cell-based therapy should be validated in a scaled-up, physiologically pertinent large animal model whenever possible. Regardless of the advantages of small animals, several features of human cardiovascular physiology cannot be reproduced in these animal models. For instance, recognition of pacemaker activity in stem cell transplants could be prevented by the high heart rate of mice or rats, while implanting the same cells into a larger animal model such as dogs, pigs, or sheep could allow the detection of such complications [146].

Although the exact mechanisms of MSCs therapy are not well defined, prosperity of preclinical studies showing the feasibility, efficacy, and safety of such therapy as mentioned above paved their way to enter the clinical trials for human cardiac regeneration. However, variations among different laboratories in using diverse sources of tissues, methods of extraction, protocols for culturing, and tools for characterization led to many debates about the characteristics and potencies of MSCs. These disparities may cause isolation and expansion of distinct subpopulations of cells or may alter the cell characteristics [147]. For example, comparing MSCs cultivated in human supplements to those cultured in fetal bovine serum (FBS) demonstrated that FBS modifies the expression of genes involved in differentiation and adhesion/extracellular matrix to some extent [50, 147]. Furthermore, insufficiency of MSCs regularly necessitates *ex vivo *expansion; however, widespread expansion may result in futile or collapsed cells [148]. 

Further, clinical trials using MSCs that are obtained and characterized by a number of diverse protocols may limit the reproduction or the elucidation of the clinical findings [149]. Therefore, the challenge for researchers intending to produce MSCs for clinical trials is to delineate the finest cell culture conditions for efficient isolation and *ex vivo *expansion of homogenous MSCs along with maintaining the cellular characteristics needed for the planned clinical application and diminishing possibilities of undesirable side effects at the same time [50]. This requires that the whole MSC manufacturing process from starting material until potency testing for the planned clinical application should be extremely standardized to obtain the required and reproducible cellular characteristics and potencies [50]. In this regard, using adult stem cell types in clinical studies, normally, needs formal approval by the respective regulatory body. This approval entails that cellular products should be manufactured, processed, and tested according to the present national guidelines, including present good tissue practice (GTP), good manufacturing practice (GMP), and good clinical practice (GCP). Applying these regulatory frameworks on the cellular products will guarantee the safety, purity, and potency of these products and the feasibility of their use in clinical application [50].

In comparison with the BMCs, the clinical involvement of MSCs for cardiac regeneration remains in its early stages and only a few number of phase I/II clinical studies have been reported [150].  Table 2 summarizes some of the MSC clinical trials in different cardiac pathologies including MI, chronic ischemia, and heart failure. In 2004, Chen et al. [104] investigated for the first time the outcomes of intracoronary injection of autologous BM-MSCs (8–10 × 10^9^ cells) in acute MI patients. At three-month followup, marked enhancements in myocardial perfusion, LV ejection fraction, and LV chamber dimensions were evident in MSC-treated patients in comparison with placebo. Notably, this paper displayed that MSC therapy is safe and devoid of deaths and arrhythmias during the follow-up period. Similarly, in 2005, Katritsis et al. [151] investigated the effect of a combination of intracoronary transplantation of BM-derived MSCs and EPCs (2–4 × 10^6^ cells) on tissue repair in myocardial scars of patients with an anteroseptal MI. At four-month followup, they reported a significant decrease in wall motion score index and significant increases in myocardial viability and contractility in stem-cell treated patients compared to untreated controls. Moreover, the investigators chose only five patients into their series who had implantable cardioverter defibrillator (ICD) to examine the potential proarrhythmic effect of MSC therapy [152]. At 16–36-month followup, assessment of the ICD showed that none of the MSCs-treated exhibited either sustained or non-sustained ventricular arrhythmia. Finally, they concluded that intracoronary transplantation of MSCs and EPCs is feasible, safe, and may participate in regional myocardial regeneration following MI.

Mohyeddin-Bonab et al. [153] investigated the safety and feasibility of MSCs therapy in a pilot study of eight patients with old MI. MSCs (2.1–9.1 × 10^6^ cells) were injected either intracoronary in patients undergoing revascularization by percutaneous coronary intervention or by direct epicardial injection in patients undergoing revascularization by coronary artery bypass graft surgery. At 6–18-month followup, they revealed smaller perfusion defect, better LV ejection fraction, and enhanced heart failure functional class without reporting any adverse side effects. Therefore, MSC therapy was described to be safe and feasible in patients with old MI. In 2008, Osiris Therapeutics [146] announced the preliminary results of the first clinical trial of MSC transplantation for cardiac regeneration in the United States. Patients received allogenic MSCs transplants by intravenous infusion. At 6-month followup, MSC-treated patients exhibited improvement in the heart and lung function along with decreased arrhythmic events compared to placebo group. The researchers reported that such allogenic cell products did not necessitate prolonged cell pretreatment handlings; however they are readily accessible to fulfill the clinical community requirements [150]. A recent study by Hare et al. [103] performed a double-blind, placebo-controlled, dose-ranging (0.5, 1.6, and 5 × 10^6^ cells/kg) safety trial of intravenous allogenic MSCs in acute MI patients. Results of this study demonstrated the safety of such intervention in post-MI patients. It also demonstrated a decrease in ventricular arrhythmias, enhanced pulmonary function, and increased LV ejection fraction in MSC-treated patients after 3 months.

In 2011, Williams et al. [105] examined the functional effects of transendocardial injection of MSCs in patients with chronic ischemic cardiomyopathy secondary to MI. In this study MSC-treated patients exhibited decreased cardiac remodeling and enhanced regional contractility along with decreased end-diastolic and end-systolic volumes, at 3 months following stem cell injection and continued up to one year. Notably, there was no evidence of ectopic tissue growth or sustained arrhythmias at one year after transplantation. This data indicates not only the safety of MSC therapy for post-MI transplantation but also the efficacy of such therapy in modulating cardiac structure and function. Most recently, Bartunek et al. [154] reported the results of the C-CURE clinical trial for the treatment of ischemic cardiomyopathy. In this study, guided cardiopoietic-MSC therapy was delivered to viable but defective myocardium by electromechanical guidance. At 6-month followup, the results showed significant enhancements in clinical performance and ejection fraction, compatible with improvement in end-diastolic and end-systolic volumes in cardiopoietic-MSC therapy group compared to controls. Importantly, evidence of cardiopoietic MSC-induced arrhythmias or toxicity was absent.

Furthermore, a number of other clinical trial efforts are on track. Consistent with the registered data from http://www.clinicaltrials.gov, a web-based service by the National Institutes of Health of the United States, there are ten ongoing phase I/II trials to evaluate the efficacy and/or safety of MSCs therapy for cardiac regeneration in diverse places in the world including the United States, Europe, and East and South Asia (Table 3). These studies are using different interventions for applying autologous and/or allogenic MSCs in the treatment of different cardiac pathologies such as acute MI, chronic ischemic LV dysfunction secondary to MI, and nonischemic dilated cardiomyopathy.

## 5. Future Perspectives of Mesenchymal Stem Cell Therapy

Overall, preclinical and clinical data from animal models and humans have demonstrated the feasibility, safety, and efficiency of MSCs therapy for cardiac regeneration. Accordingly, MSC therapy assures myocardial repair for a large number of heart failure patients; yet, there are several aspects that still need to be resolved. This will need rigorous investigation in the years to come [53]. Future studies should focus on the efficiency of MSC therapy in animals at different ages (adult and old), instead of young adult animals only. Investigating the efficacy of MSC treatment combined with standard post-MI therapies, such as angiotensin converting enzyme inhibitors and beta-blockers, is also necessary to maximize the therapeutic benefits. Subsequently, dose escalation studies will be required to optimize MSC therapy before being considered as a potential clinical treatment. It is also important to consider the potential benefits of MSC therapy in nonischemic heart failure models rather than the commonly used post-MI model [53]. Approaches to improve engraftment and differentiation are required due to the low retention of cardiac stem cells regardless of the delivery method used. Moreover, the precise mechanism of action of MSCs needs to be specifically defined; it is still not clear if they work through paracrine signaling, cell fusion, cell-cell interaction, differentiation to cardiomyocytes, neovascularization, and/or a combination of some or all of these effects.

## Figures and Tables

**Table 1:** Effects of MSC therapy on both small and large animal models of MI. MI, myocardial infarction; DI, direct intramyocardial injection; IV, intravenous infusion; IS, in situ injection; TESI, transendocardial stem cell injection; IC, intracoronary infusion; LV, left ventricular; EF, ejection fraction; ESV, end-systolic volume; EDV, end-diastolic volume; ↑, increase; ↓, decrease. *The monolayered cell graft was placed on a plastic sheet and applied face down onto the surface of the infarct area. The plastic sheet was then carefully removed, leaving the monolayered cell graft on the surface of the heart.

Species/model	Dose	Results	Reference
Mice			
Acute MI	0.5–5 × 10^5^ (DI)	↓ Both infarct size and fibrosis at 2 weeks	Kudo et al. [68]
Acute MI	3 × 10^5^ (DI)	↑ Cardiac function at 4 weeks	Fazel et al. [65]
Acute MI	5 × 10^5^ (DI)	↓ Infarct size; ↑ cardiac function at 3 days	Noiseux et al. [70]
Acute MI	1 × 10^6^ (DI)	↑ LVEF at 2 and 4 weeks	Nakamura et al. [69]
Acute MI	1 × 10^6^ (DI)	↑ Cardiac function at 1 month	Shiota et al. [71]
Acute MI	2 × 10^5^ (DI)	↑ LVEF and LV function at 2 weeks	Grauss et al. [67]

Rats			

Acute MI	5 × 10^6^ (DI)	↓ Cardiac remodeling; ↑ cardiac performance at 2 weeks	Mangi et al. [77]
Acute MI	5 × 10^6^ (IV)	↑ Cardiac function; ↓ infarct size at 4 weeks	Nagaya et al. [79]
Acute MI	2 × 10^6^ (DI)	Transient global LV function improvement at 4 weeks	Dai et al. [73]
Acute MI	2 × 10^6^ (DI)	↓ Fibrosis; ↑ cardiac function at 8 weeks	Berry et al. [72]
Acute MI	Cell graft*	Reversed wall thinning; ↑ cardiac function at 8 weeks	Miyahara et al. [78]
Acute MI	6 × 10^6^ (DI)	↑ LVEF; ↓ infarct size at 3 weeks	Li et al. [76]
Acute MI	1 × 10^6^ (IS)	↑ LVEF; ↓ infarct size at 30 days	de Macedo Braga et al. [74]
Acute MI	5 × 10^6^ (DI)	↑ LVFS; ↓ fibrosis at 4 weeks	Imanishi et al. [75]

Swine			

Subacute MI	6 × 10^7^ (DI)	↓ Wall thinning in the scar area; ↑ cardiac function at 4 weeks	Shake et al. [92]
Acute MI	2 × 10^8^ (TESI)	↓ Necrotic myocardium; ↑ cardiac performance over 8 weeks	Amado et al. [80]
Chronic MI	2 × 10^8^ (DI)	Preserved LVEF at 60 and 90 days post-MI	Makkar et al. [86]
Acute MI	2 × 10^8^ (TESI)	↓ Infarct size at 1 and 8 weeks; restored contractile function	Amado et al. [81]
Acute MI	3.2 × 10^8^ (IV)	↑ LVEF; ↓ hypertrophy at 3 months	Price et al. [87]
Subacute MI	6.3 × 10^5^ (TESI)	↓ Scar size; ↓ EDV; ↑ LVEF at 10 days	Gyongyosi et al. [82]
Chronic MI	1–10 × 10^6^ (IV)	↑ Vasculogenesis; ↑ regional perfusion; no change in LVEF at 12 weeks	Halkos et al. [83]
Acute MI	0.24–4.4 × 10^8^ (TESI)	↓ Scar size; no change in LVEF at 12 weeks	Hashemi et al. [84].
Acute MI	1 × 10^7^ (IC)	↑ EF; ↓ scar size at 8 weeks	Qi et al. [88]
Acute MI	2 × 10^8^ (TESI)	↑ Myocardial blood flow at 1 week; ↑ LV function at 8 weeks	Schuleri et al. [90]
Chronic MI	2 × 10^8^ (TESI)	↓ Scar size; ↑ EF; ↑ regional contractility; ↑ myocardial perfusion over 12 weeks	Quevedo et al. [89]
Chronic MI	0.2–2 × 10^8^ (DI)	↓ Scar size; ↑ EF; ↑ regional contractility; ↑ myocardial perfusion at 12 and 24 weeks	Schuleri et al. [91]
Acute MI	0.75–1 × 10^8^ (TESI)	↓ Scar size; ↑ EF at 2 and 8 weeks	Hatzistergos et al. [85]

Canine			

Chronic ischemia	1 × 10^8^ (DI)	↓ Fibrosis; ↑ LVEF at 60 days	Silva et al. [94]
Subacute MI	1 × 10^8^ (IC/TESI)	↑ EF; ↓ myocardial ischemia; ↓ EDV and ESV at 21 days post-MI	Perin et al. [93]

Sheep			

Acute MI	25–450 × 10^6^ (DI)	↓ Infarct expansion; ↑ vascular density in the border zone; ↑ EF; ↓ EDV at 8 weeks	Hamamoto et al. [95]

**Table 2:** MSC clinical trials in MI, chronic ischemia, and heart failure. MI, myocardial infarction; IC, intracoronary infusion; DI, direct intramyocardial injection; IV, intravenous infusion; TESI, transendocardial stem cell injection; EMG; electromechanical guidance; LV, left ventricular; EF, ejection fraction; ESV, end-systolic volume; EDV, end-diastolic volume; ↑, increase; ↓, decrease.

Group	Condition	Dose (cells)	Followup (months)	Results
Chen et al. [104]	Acute MI	8–10 × 10^9^ (IC)	3	↑ Myocardial perfusion, ↑ LVEF, and ↓ LV chamber dimensions
Katritsis et al. [151]	Anteroseptal MI	2–4 × 10^6^ (IC)	4	↓ Wall motion score index and ↑ myocardial viability and contractility
Mohyeddin-Bonab et al. [153]	Old MI	2.1–9.1 × 10^6^ (IC)/(DI)	6–18	↓ Perfusion defect and ↑ LVEF
Osiris therapeutics [146]	Acute MI	(IV)	6	↑ Heart function and ↓ arrhythmic events
Hare et al. [103]	Acute MI	0.5, 1.6, and 5 × 10^6^ (IV)	3	↑ LVEF and ↓ ventricular arrhythmia
Williams et al. [105]	Chronic ischemic cardiomyopathy secondary to MI	10 repeated injections of 0.5 mL of cell suspension (TESI)	3–12	↓ Cardiac remodeling, ↓ ESV and EDV, and ↑ regional contractility
Bartunek et al. [154] (C-CURE)	Heart failure secondary to ischemic cardiomyopathy	6–12 × 10^8^ (EMG)	6	↑ LVEF and ↓ ESV and EDV

**Table 3:** Ongoing clinical trials on MSCs: condition, intervention/dose, and followup in patients around the world (http://www.clinicaltrials.gov).

World	Condition	Intervention	Time frame	Phase/Status
Florida (USA)	Chronic ischemic LV dysfunction secondary to MI	10 and 20 intramyocardial injections of 2 million MSCs (low dose) or 20 million (high dose)/0.25–0.5 cm^3^ for a total of 20 million or 200 million cells, respectively	6–18 months	Phase I/II (unknown)
	Chronic ischemic LV dysfunction and heart failure secondary to MI	Transendocardial injection of autologous human cells (bone marrow or mesenchymal). 40 million cells/mL delivered in either a dose of 0.25 mL/injection for a total of 100 million × 10 injections or a dose of 0.5 mL/injection for a total of 200 million × 10 injections	6–18 months	Phase I/II (unknown)
	Chronic ischemic LV dysfunction secondary to MI	Transendocardial injection of autologous versus allogeneic MSCs. 40 million cells/mL delivered in either a dose of 0.5 mL/injection × 1 injection for a total of 20 million, a dose of 0.5 mL/injection × 5 injections for a total of 100 million, or a dose of 0.5 mL/injection × 10 injections for a total of 200 million MSCs	6–13 months	Phase I/II (active)
	Nonischemic dilated cardiomyopathy	Transendocardial injection of autologous versus allogeneic MSCs. 20 million cells/mL delivered in a dose of 0.5 mL/injection × 10 injections for a total of 100 million of MSCs	6–12 months	Phase I/II (active)

Maryland (USA)	Chronic ischemic LV dysfunction secondary to MI	10 and 20 intramyocardial injections of 2 million MSCs (low dose) or 20 million (high dose)/0.25–0.5 cm^3^ for a total of 20 million or 200 million of autologous human MSCs, respectively	6–18 months	Phase I/II (unknown)
	Chronic ischemic LV dysfunction secondary to MI	Transendocardial injection of autologous versus allogeneic MSCs. 40 million cells/mL delivered in either a dose of 0.5 mL/injection × 1 injection for a total of 20 million, a dose of 0.5 mL/injection × 5 injections for a total of 100 million, or a dose of 0.5 mL/injection × 10 injections for a total of 200 million MSCs	6–13 months	Phase I/II (active)

France (Europe)	Chronic myocardial ischemia; LV dysfunction	Transendocardial intramyocardial injections of 60 million autologous MSCs	30 days–2 years	Phase I/II (active)

China (East Asia)	ST-elevation MI	Intracoronary human umbilical WJ-MSC transfer	4 months–1 year	Phase II (active)

Korea (East Asia)	Acute MI	Intracoronary injection of single dose of autologous bone-marrow-derived MSCs (I million) cells/kg	6 months	Phase II (completed)

India (South Asia)	ST-elevation acute MI	A Single Dose of Intravenous infusion of Allogenic MSCs	6 months	Phase I/II (active)
